# The effect of age-related decline in physiological activity on the concealed information test

**DOI:** 10.3389/fpsyg.2025.1733181

**Published:** 2026-01-28

**Authors:** Robin Orthey, Yoshiyuki Tsuyama, Izumi Ikeda, Takahiro Yoshioka, Masaki Ishihara, Izumi Matsuda

**Affiliations:** 1 Converging Technology Fundamental Project, Fujitsu Laboratories, Kawasaki, Japan; 2 Department of Psychology, Aoyama Gakuin Daigaku, Shibuya, Japan

**Keywords:** age, concealed information test (CIT), heart rate, respiration, skin conductance response (SCR)

## Abstract

A demographic shift towards aging societies necessitates a reexamination of established psychological tests with age-related decline of physiological responsivity in mind. The Concealed Information Test (CIT), a physiological test to detect memory of crime details, is frequently and effectively used in criminal investigations in Japan, a severe case of an aging society. Its validity has been well established, but almost all the support stems from data of young and healthy university students. Yet, the age-related decline in physiological activity is well documented, but it is unclear how the CIT is affected. We examined the robustness of the CIT to the effects of advanced age by subjecting 33 seniors, aged 62-80, to a standard CIT examination, measuring skin conductance response, heart rate, and respiration over two conditions. Once participants were aware of secret information and hid it, and once they were ignorant of the target piece of information. Skin conductance response was an effective measure, but the proportion of non-responders was higher than expected. Heart rate was not diagnostic with the standard transformation procedure, but with a -1-0s baseline correction the test could detect hidden knowledge. Respiration was not diagnostic. Our findings suggest that all three CIT indices are affected by age. Therefore, while modifications to data analysis can partially mitigate age-related effects on CIT accuracy, the overall reduction in physiological indices warrants a cautious approach when faced with senior examinees and highlights the need for further research into age-related physiological changes impacting applied tests such as the CIT.

## 1 Introduction

Harper (2014) projects that by 2050 senior and young citizens will make up the same proportion of the population. In the context of rapidly aging populations across developed societies (e.g., D'Ambrogio, 2020), it is crucial to consider the impact of age-related decline in physiological responsivity on psychological testing. Despite this demographic trend, most psychological research is still conducted with young university students (Arnett, 2016), whose physiological profile is unlikely to represent the broader population. This discrepancy raises the critical question whether established tests that were validated in a young and healthy population remain diagnostic for examinees with reduced physiological responsivity due to, for example, age. A prime example of this situation is the Concealed Information Test (CIT), a test to detect familiar stimuli, usually representing crime details, based on indices of the autonomic nervous system. Despite being based on a strong scientific foundation, the effects of age-related decline remain unexplored. Here, we seek to test if the CIT remains diagnostic for examinees of advanced age.

The CIT is commonly used in Japan to identify suspects who know more about a crime than they are willing to admit (Osugi, 2011). It is based on a common research paradigm in psychophysiology, the oddball paradigm. In a typical oddball paradigm, a subject is presented with a series of stimuli one after another. All stimuli are alike except for one, the oddball, which evokes an elevated physiological response (e.g., Squires et al., 1975). The CIT uses the same phenomenon to identify examinees who are concealing their knowledge of crime details. Each question about the crime is paired with four to six answers. All answers should be plausible, but only one must be correct. The CIT is diagnostic, because the correct answer only stands out to those who know it is correct (Lykken, 1974).

Since its inception the CIT has received strong support from the scientific community. Two meta-analyses (Ben-Shakhar and Elaad, 2003; Meijer et al., 2014) demonstrate strong effect sizes for the standard CIT measures, i.e., Skin Conductance Response (SCR), Heart Rate (HR) and Respiration (RESP). Furthermore, Zaitsu (2016) compared CIT examinations produced in psychological research with real CIT examinations of suspects and demonstrates that CIT research represents findings in practice well. However, a limitation of the CIT literature is that it is based on young university students, a widespread problem in psychology (Arnett, 2016; Peterson, 2001). To our knowledge, there is only one study that directly examined differences across age. Sauerland et al. (2025) used a response time-based CIT for familiar face detection and found the effectiveness of the CIT to be stable across age groups. However, the oldest age range for the CIT in this study was 36 to 60 years and no physiological responses were measured. Hence, despite the wealth of support for the CIT’s validity, the effects of old age remain unclear.

There may be no direct evidence of the effects of age on the CIT’s validity, but there is a large body of evidence that has examined how age affects the three main measures the CIT relies on, respiration, heart rate, and electrodermal activity. For example, while maintaining general functionality, respiratory muscle strength, lung function, and exercise capacity decrease with age (Sharma and Goodwin, 2006; Watsford et al., 2007). A similar pattern has been reported for heart rate. Resting levels of heart rate maximum heart rate and heart rate variability gradually decline from adulthood (Kerkhof et al., 2018; Quigley et al., 2024). Furthermore, participants of advanced age show lower heart rate reactivity than their younger counterparts when experiencing emotionally loaded stimuli (Uchino et al., 2010). In the case of EDA, the consensus is that it declines with age, too (Boucsein, 2012). However, the findings are not as consistent as for respiration and heart rate. For example, Gavazzeni et al. (2008) found a clear difference between 20–30- and 70–80-year-olds. Despite rating negative stimuli as more intense, older participants exhibited more attenuated electrodermal activity compared to young participants when viewing negative stimuli. Similarly, Zelinski et al. (1978) found that older participants demonstrated lower SCRs in a memory test than younger participants. However, in some cases no relationship between age and EDA was found. For example, Garwood et al. (1979) reported no influence of age on SCR in a reaction time task. Furchtgott and Busenmeyer (1979) also found no age group differences in math and memory tasks. In sum, there is considerable evidence linking age with reduced responsivity of the autonomic nervous system.

The findings of previous studies highlight age-related decline in responsivity of the autonomic nervous system, that consequently may lead to a reduced effectiveness of the CIT in elderly subjects. Our goal is to examine the robustness of the CIT to these effects of advanced age. We subject a sample of senior citizens from the general population to two standard CIT examinations. Once as a suspect with hidden information and once without. We record the standard CIT measures, skin conductance response (SCR), heart rate (HR), and respiration (RESP) and examine their diagnostic validity. We expect that all three indices will detect concealed knowledge. Specifically, we expect larger SCR, shorter HR, and shorter RESP when examinees are aware of the correct answer (H1). We also expect this difference to have a better diagnostic accuracy than chance performance, i.e., to feature an Area Under the Curve larger than 0.5 (H2).

## 2 Method

This experiment was preregistered here: https://osf.io/kjc56/?view_only=73ccbbdcb9d2417783f5846046372eb1 (https://osf.io/ybkqv/registrations). We deviated from the preregistration in two aspects. Firstly, we planned to assess an additional remote sensing device. However, the recorded data with this device was unusable, so all analyses regarding the remote sensing aspect have been left out. Secondly, we intended to include a CIT question about a place the examinee had visited over 10 years ago. Examinees were meant to submit some relevant information via letter in advance, but more than half failed to do so due to an error at the recruitment company. Hence, we omitted posing a third CIT question. Other amendments to the data transformation are mentioned where they occur.

### 2.1 Participants

A sample of 33 senior citizens were recruited for this study. Participants were approached by a recruitment agency they registered at voluntarily. Two were excluded due to technical problems with the recording setup. From the remaining sample we excluded three participants due to excessive noise in their responses for heart rate. Leaving a final sample of 28 participants with 19 men and nine women. Participants’ age ranged from 62 to 80 years with a mean age of 70.82 (SD = 3.95). All participants were native Japanese speakers. We sought to only recruit participants who were in good health, so participants who exhibited one or more of the following conditions were not approached during recruitment: any heart disease/problem; use of a pacemaker; any lung disease/problem; history of chain smoking; any memory problem (e.g., Alzheimer’s), being deaf or blind; being unable to sit for 30 min without getting up; any use of pain medication; being above the age of 80. Furthermore, participants were excluded for the following reasons: technical faults in the recording equipment; not following the experimenters’ instructions; not remembering the correct answer during the debrief. The data of one participant was excluded due to errors during recording. Participants received 3.000JPY for participating and ethical approval was obtained from Aoyma Gakuin University (H24-13).

### 2.2 Procedure

The experiment took place in a climatized room, and participants were informed about the purpose and procedure of the experiment before signing an informed consent form. Next participants were attached to the physiological recording equipment, and we recorded a two-minute baseline. Then the CIT examination began. We subjected participants to the standard card test procedure twice, either with odd (1, 3, 5, 7, 9) or even (2, 4, 6, 8, 10) numbers as done previously in Matsuda et al. (2009). For one question, participants drew a card with one of the numbers from the set and were instructed to memorize and conceal their knowledge of this number. For the other, a random card was set aside and designated to be the correct answer, but examinees were unaware which number it was. All participants received both questions in a counterbalanced fashion. Both times the procedure of the card test was the same. The CIT question, “What is the number of the set aside card?,” was followed by five repetitions of the five possible answer alternatives. The sequence of answer alternatives during each repetition was determined randomly and there was an Inter Stimulus Interval of 21–23 s between stimuli. Each time an answer was presented, the examinee had to verbally deny it by saying “No.” After both CIT questions, we confirmed that participants could remember the number on their set aside card. Failure to remember the number would have resulted in exclusion, but all examinees did correctly recall it.

### 2.3 Data transformation

The experiment featured a 2 veracity (concealed knowledge vs. no concealed knowledge) within subject design with SCR, heart rate, and respiration as dependent variables. EDA and heart rate were recorded with the Polymate system (AP108, TEAC, Japan) and recorded at a 1,000 Hz sampling rate.

Electrodermal Activity (EDA). EDA was measured using a 0.5-V constant voltage system. Two Ag/AgCl disposable electrodes containing 0.05 M NaCl electrolyte (PPS-EDA, TEAC, Japan) were attached to the volar side of the distal phalanges of the second and third fingers of the non-dominant hand. EDA data was offline downsampled to 100 Hz. We defined the SCR as the maximum positive increase in the EDA waveform in the time window of 1–5 s following stimulus presentation. If EDA only declined in a trial, the value for this trial was set to 0. In line with Klein Selle et al. (2019) participants were classified as non-responders if the standard deviation over the first and last five answers was smaller than 0.01 μS.

Heart rate (HR). Finger pulse volume was recorded using a near-infrared light-emitting diode (810 nm), and a photodiode was attached at opposite sides of the distal phalanges of the fourth finger of the non-dominant hand. This pulse wave was used to compute the heart rate, by identifying peak-to-peak intervals (IBI). HR was defined as the average IBI over a specified period. We defined three intervals of interest based on a field study conducted by CIT practitioners (Kobayashi et al., 2009) and researchers connected to the institute governing the CIT practice in Japan (Hirota et al., 2009). We examined the 0–10 s, 10–20 s, and 0–20 s periods following stimulus presentation.

Respiration Line Length (RLL). We preregistered to calculate the RLL according to Matsuda and Ogawa (2011), however due to the poor breathing quality of elderly participants it was difficult to clearly mark the beginning and ends of breath cycles. Therefore, we followed the standard CIT methodology (Timm, 1982) and calculated the RLL as the total distance in the 5–15 s interval following stimulus presentation.

All dependent variables were calculated as described above. There were five sets of answers, and each set was repeated five times per condition. Among the five unique answers one was correct, and the others were not. In the CIT we examine the response to the correct answer relative to the incorrect answers, therefore we standardized the responses according to Ben-Shakhar (1985). For each repetition we computed the mean and standard deviation of all five answer alternatives and standardized the response to the correct answer using this mean and standard deviation. We did this for all five repetitions and then averaged the standardized response to the correct answer. This results in a single value per dependent variable per condition for each participant.

Detection accuracy will be assessed with the Area Under the Curve (AUC) of the Receiver Operating Characteristic (ROC; Hanley and McNeil, 1982). The AUC ranges between 0 and 1, with 0.5 representing chance performance. It is a method to assess the diagnostic accuracy of an index without committing to a specific decision threshold. The ROC computes the sensitivity, the correct classification rate of concealed knowledge, and specificity, the correct classification rate of the absence of concealed knowledge, over all cut-off thresholds and thus is a suitable general measure of detection accuracy. AUCs significantly larger than 0.5 suggest that the index has diagnostic value.

## 3 Results

### 3.1 Physiological responses

#### 3.1.1 Heart rate

**Figure 1:**
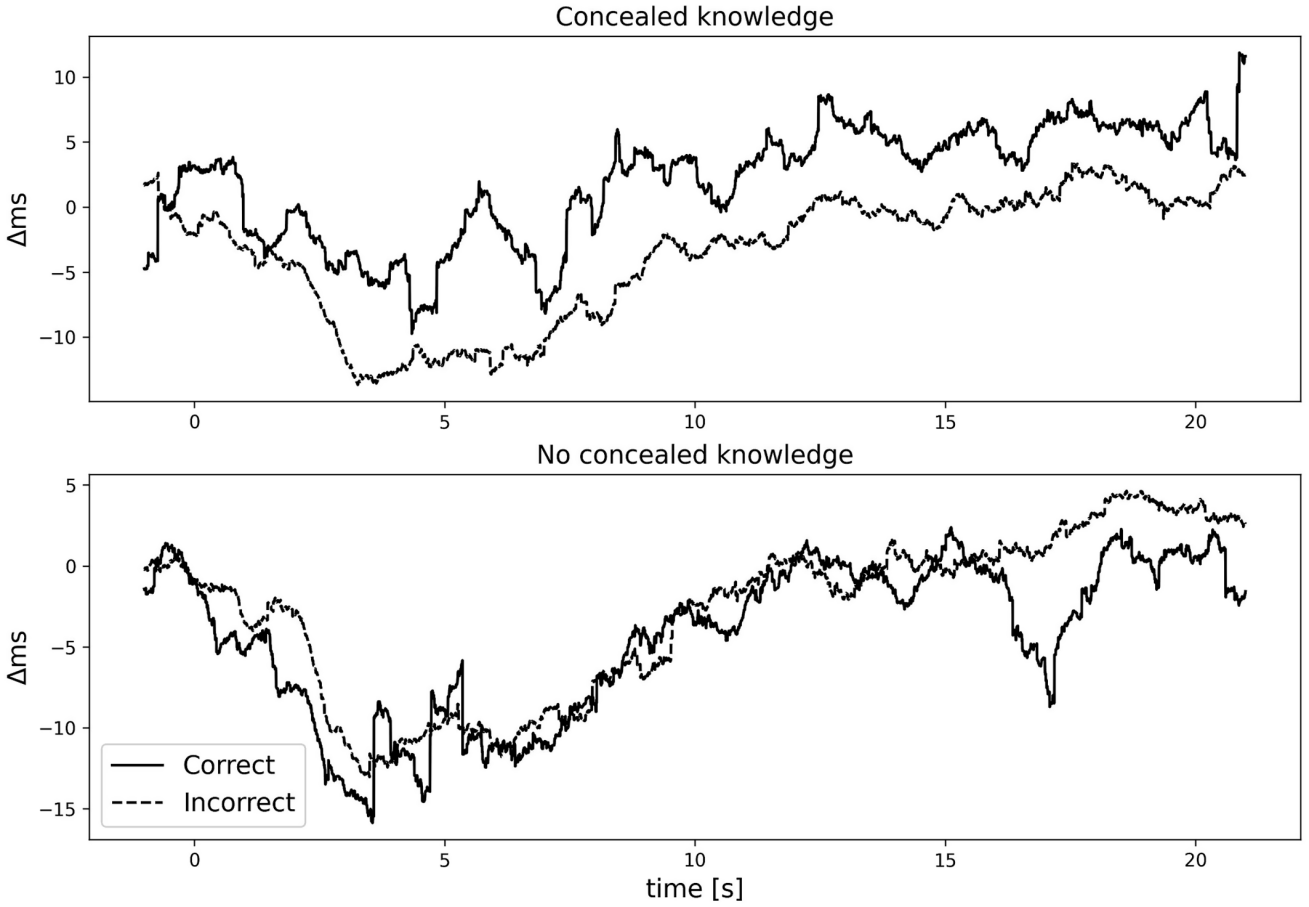
Grand average of baseline corrected IBI time-course for correct and incorrect answers in the concealed knowledge and no concealed knowledge conditions.

The time course of the IBI grand average for correct and incorrect answers in both conditions can be seen in Figure 1. Typically, HR is assessed in the 10–20s period in the CIT. However, we noticed that the 0–10s period could be of interest, so we included the 0–10s and 0–20s intervals as regions of interest, too. We conducted a paired-samples *t*-test between the concealed knowledge and no concealed knowledge condition with HR as the dependent variable. We examined the 0–10s, 10–20s, and 0–20s intervals. There was no significant difference for the 0–10s interval, *t*(27) = 1.40, *p* = *0*.173, Cohen’s *d* = 0.27, the 10–20s interval, *t*(27) = 0.66, *p* = *0*.513, Cohen’s *d* = 0.13,or the 0–20s interval, *t*(27) = 1.07, *p* = *0*.296, Cohen’s *d* = 0.20. In all cases, HR did not differ between conditions (0–10s: *m_concealed knowledge_* = 0.19, *SD_concealed knowledge_* = 0.54, *m_no concealed knowledge_* = 0.009, *SD_no concealed knowledge_* = 0.33; 10–20s: *m_concealed knowledge_* = 0.11, *SD_concealed knowledge_* = 0.49, *m_no concealed knowledge_* = 0.03, *SD_no concealed knowledge_* = 0.49; 0–20s: *m_concealed knowledge_* = 0.17, *SD_concealed knowledge_* = 0.44, *m_no concealed knowledge_* = 0.03, *SD_no concealed knowledge_* = 0.45).

After inspecting the individual epochs and grand average, we noticed that responses to the stimuli persisted longer than 20 s and therefore could affect the response of the following stimulus. To account for a potential delay in the natural return to baseline, we applied a −1 – 0 s baseline correction to examine ΔHR and ran the analyses again. There was a significant difference for the 0–10s interval, *t*(27) = 3.44, *p* = *0*.002, Cohen’s *d* = 0.65. There was also a significant difference for the 10–20s interval, *t*(27) = 2.47, *p* = 0.02, Cohen’s *d* = 0.47. The 0–20s interval was significant, too, *t*(27) = 3.09, *p* = 0.005, Cohen’s *d* = 0.58. In all cases, HR was lower (resulting in a larger standardized score) in the concealed knowledge condition (0–10s: *m_concealed knowledge_* = 0.36, *SD_concealed knowledge_* = 0.44, *m_no concealed knowledge_* = −0.03, *SD_no concealed knowledge_* = 0.50; 10–20s: *m_concealed knowledge_* = 0.28, *SD_concealed knowledge_* = 0.47, *m_no concealed knowledge_* = 0.0003, *SD_no concealed knowledge_* = 0.44; 0–20s: *m_concealed knowledge_* = 0.34, *SD_concealed knowledge_* = 0.44, *m_no concealed knowledge_* = 0.004, *SD_no concealed knowledge_* = 0.49).

#### 3.1.2 Respiration

We conducted a paired samples *t*-test between the concealed knowledge and no concealed knowledge condition with average RLL as dependent variable. There was no significant difference, *t*(27) = −0.81, *p* = 0.427, Cohen’s *d* = −0.15, between the concealed knowledge condition, *m* = −0.25, *SD* = 0.47, and the no concealed knowledge condition, *m* = −0.15, *SD* = 0.43.

#### 3.1.3 Skin conductance response

In line with Klein Selle et al. (2019) we first identified non-responders. In our sample, no participants were non-responders. However, nine participants passed this classification but showed no response at all in at least one condition, resulting in a non-numeric value during the standardization procedure. With the remaining sample of 19 participants, we conducted a paired samples *t*-test between the concealed knowledge and no concealed knowledge condition with SCR as the dependent variable. There was a significant difference between conditions, *t*(18) = 2.74, *p* = 0.013, Cohen’s *d* = 0.63. Participants showed a larger SCR when they were hiding their knowledge, *m* = 0.61; *SD* = 0.54, than when they were unaware of the correct answer, *m* = 0.11; *SD* = 0.48.

Only HR and in some cases, SCR exhibited differences between concealed knowledge and no concealed knowledge, so H1 is only partly supported.

### 3.2 Detection accuracy

We also calculated the Area Under the Curve (AUC) to assess the diagnostic value of all measures. The results can be seen in Table 1. We found that SCR and HR, all intervals, were diagnostic. Only RLL had no diagnostic value. We omit the preregistered analysis of combining all measures, because SCR is based on fewer participants and RLL was not effective. Only HR and SCR were diagnostic CIT criteria, so H2 is only partly supported.

**Table 1:** Diagnostic accuracy of SCR, HR, and RLL.

Measures	AUC	*p*	95% CI
SCR	0.75	0.013*	[0.55–0.94]
RLL	0.44	0.492	[0.29–0.60]
HR			
0–10s	0.73	<0.001**	[0.60–0.87]
10–20s	0.66	0.026*	[0.52–0.81]
0–20s	0.68	0.009*	[0.55–0.83]

*p < 0.05; **p < 0.001.

## 4 Discussion

A demographic shift towards aging societies and the disproportional use of young university students in psychological research necessitates a reexamination of psychological tests with the effects of advanced age in mind. It is known that age-related decline affects the physiological measures used by the CIT, but despite a wealth of studies supporting the validity of the CIT, we do not know how it is impacted by age. We subjected senior citizens to a standard CIT examination. Once they knew the correct answer and had to hide it, and once without knowing which answer was correct, while we measured electrodermal activity, heart rate and respiration. We expected that each measure would differ significantly contingent on the participant’s knowledge of the correct answer and that each measure would be a diagnostic criterion. However, our hypotheses were only partly supported.

We found that all three standard CIT measures were affected by age-related decline in physiological activity. In line with prior findings of age-related decline in respiratory muscle strength (Sharma and Goodwin, 2006; Watsford et al., 2007), we found respiration to be ineffective (AUC = 0.44) which stands in stark contrast to the expectation from the literature (AUC = 0.77; Meijer et al., 2014). Similarly, when using the standard CIT transformation procedure heart rate was not diagnostic. Only when we applied a one-second baseline correction, heart rate featured a strong effect (AUC 0–10s = 0.73; AUC 10–20s = 0.66; AUC 0–20s = 0.68), comparable to other studies in the CIT literature (AUC = 0.74; Meijer et al., 2014). We noticed that there was no consistent respiration pattern and that heart rate levels varied within participants over the course of the CIT examination. This variation could explain why both measures were ineffective using the standard CIT transformation procedure. Similarly, our results for skin conductance response reflect the mixed findings in the literature on electrodermal activity in aging. Some studies report diminished responsivity in the elderly (Gavazzeni et al., 2008; Zelinski et al., 1978), while others did not find any differences (Garwood et al., 1979; Furchtgott and Busenmeyer, 1979). We found that there was an unusually high number of participants who showed no responses to any of the items, resulting in a smaller than preregistered sample size for SCR. The smaller sample size warrants additional caution when interpreting the SCR results, but SCR is the strongest CIT measure and those who did respond to the stimuli showed a strong effect (AUC = 0.75), although not as strong as would be expected from the CIT literature (AUC = 0.85; Meijer et al., 2014). Additionally, it is important to note that we used strict selection criteria when recruiting seniors, which means our participants were healthier than the typical individuals who take the CIT. As a result, our findings provide a conservative estimate of the negative effects of aging. Thus, under the right conditions, electrodermal activity and heart rate can serve as diagnostic measures for the CIT in elderly participants. However, respiration is not effective for this purpose and more research is needed to make it diagnostic.

Our findings should concern practitioners. CIT examiners make their judgements by considering all the indices collectively (Osugi, 2011). Although the early period following stimulus presentation, a period practitioners tend to avoid analyzing because it coincides with subjects’ verbal responses, was diagnostic, heart rate in general was only diagnostic when we applied a baseline correction procedure. Electrodermal activity, despite being effective, was frequently unavailable. Respiration was entirely ineffective. Consequently, practitioners faced with elderly CIT examinees often have to rely on only a reduced portion of the typical data when deciding. Whether the inclusion of the early period for HR can meaningfully improve this process, remains to be seen. It is therefore important to consider that advanced age can be a factor affecting data quality. Follow-up research is needed to examine whether practitioners can account for this loss of quality in their decision-making process and if the CIT procedure can be adapted to reduce the effect of age.

The CIT was re-evaluated to account for the effects of advanced age. The well-documented decline in physiological activity due to aging impacts the three standard measures used in the CIT, and not all effects can be corrected for. Consequently, practitioners may have to rely on a reduced amount of data when making their judgments. We recommend exercising caution when evaluating senior citizens and urge further research into how age affects the validity of the CIT.

## Data Availability

The dataset used in this study is available on the Open Science Framework: https://osf.io/ybkqv/overview.

## References

[ref1] Arnett J. J. (2016). “The neglected 95%: why American psychology needs to become less American” in Methodological issues and strategies in clinical research. ed. Kazdin A. E.. 4th ed (Washington: American Psychological Association), 115–132.

[ref2] Ben-Shakhar G. (1985). Standardization within individuals: a simple method to neutralize individual differences in skin conductance. Psychophysiology 22, 292–299. doi: 10.1111/j.1469-8986.1985.tb01603.x, 4011799

[ref3] Ben-Shakhar G. Elaad E. (2003). The validity of psychophysiological detection of information with the guilty knowledge test: a meta-analytic review. Psychology 88, 131–151. doi: 10.1037/0021-9010.88.1.131 12675401

[ref4] Boucsein W. (2012). Electrodermal activity. 2nd Edn. New York: Springer Science + Business Media.

[ref5] D'Ambrogio Enrico (2020). Japan's ageing society. European Parliament. Archived (PDF). Available online at: https://www.europarl.europa.eu/RegData/etudes/BRIE/2020/659419/EPRS_BRI(2020)659419_EN.pdf (Accessed October 10th 2025)

[ref6] Furchtgott E. Busenmeyer J. K. (1979). Heart rate and skin conductance during cognitive processes as a function of age. J. Gerontol. 34, 183–190. doi: 10.1093/geronj/34.2.183, 438472

[ref7] Garwood M. K. Engel B. T. Quilter R. E. (1979). Age differences in the effect of epidermal hydration on electrodermal activity. Psychophysiology 16, 311–317. doi: 10.1111/j.1469-8986.1979.tb02996.x, 441225

[ref8] Gavazzeni J. Wiens S. Fischer H. (2008). Age effects to negative arousal differ for self-report and electrodermal activity. Psychophysiology 45, 148–151. doi: 10.1111/j.1469-8986.2007.00596.x, 17850240

[ref9] Hanley J. A. McNeil B. J. (1982). The meaning and use of the area under a receiver operating characteristic (ROC) curve. Radiology 143, 29–36. doi: 10.1148/radiology.143.1.7063747, 7063747

[ref10] Harper S. (2014). Economic and social implications of aging societies. Science 346, 587–591. doi: 10.1126/science.1254405, 25359967

[ref11] Hirota A. Ogawa T. Matsuda I. Takasawa N. (2009). A model of the underlying mechanism of autonomic responses in the concealed information test. Jpn. J. Physiol. Psychol. Psychophysiol. 27, 17–34. doi: 10.5674/jjppp.27.17

[ref12] Kerkhof P. L. M. Peace R. A. Macfarlane P. W. (2018). Sex- and age-related reference values in cardiology, with annotations and guidelines for interpretation. Adv. Exp. Med. Biol. 1065, 677–706. doi: 10.1007/978-3-319-77932-4_41, 30051414

[ref9001] Klein Selle N. Agari N. Ben-Shakhar G. (2019). Hide or seek? Physiological responses reflect both the decision and the attempt to conceal information. Psychological Science 30, 1424–1433. doi: 10.1177/0956797619864598, 31491366

[ref13] Kobayashi T. Yoshimoto K. Fujihara S. (2009). The contemporary situation of field polygraph tests. Jpn. J. Physiol. Psychol. Psychophysiol. 27, 5–15. doi: 10.5674/jjppp.27.5

[ref14] Lykken D. T. (1974). Psychology and the lie detector industry. Am. Psychol. 29, 725–739. doi: 10.1037/h0037441, 4451301

[ref15] Matsuda I. Nittono H. Hirota A. Ogawa T. Takasawa N. (2009). Event-related brain potentials during the standard autonomic-based concealed information test. Int. J. Psychophysiol. 74, 58–68. doi: 10.1016/j.ijpsycho.2009.07.004, 19631702

[ref16] Matsuda I. Ogawa T. (2011). Improved method for calculating the respiratory line length in the concealed information test. Int. J. Psychophysiol. 81, 65–71. doi: 10.1016/j.ijpsycho.2011.06.002, 21689693

[ref17] Meijer E. H. Klein Selle N. Elber L. Ben-Shakhar G. (2014). Memory detection with the concealed information test: a meta analysis of skin conductance, respiration, heart rate, and P300 data. Psychophysiology 51, 879–904. doi: 10.1111/psyp.12239, 24916920

[ref18] Osugi A. (2011). “Daily application of the concealed information test: Japan” in Memory detection: Theory and application of the concealed information test. eds. Verschuere B. J. BenShakhar G. Meijer E. H. (Cambridge University Press), 253–275.

[ref19] Peterson R. (2001). On the use of college students in social science research: insights from a second-order meta-analysis. J. Consum. Res. 28, 450–461. doi: 10.1086/323732

[ref20] Quigley K. S. Gianaros P. J. Norman G. J. Jennings J. R. Berntson G. G. de Geus E. J. C. (2024). Publication guidelines for human heart rate and heart rate variability studies in psychophysiology – part 1: physiological underpinnings and foundations of measurement. Psychophysiology 61:e14604. doi: 10.1111/psyp.14604, 38873876 PMC11539922

[ref21] Sauerland M. Wiechert S. Czarnojan E. Deiman E. Dörr L. Broers N. J. . (2025). Identification performance across the life span: lineups and the reaction time-based concealed information test. Cognition 254:105996. doi: 10.1016/j.cognition.2024.105996, 39520936

[ref22] Sharma G. Goodwin J. (2006). Effects of aging on respiratory system physiology and immunology. Clin. Interv. Aging 1, 253–260. doi: 10.2147/ciia.2006.1.3.253, 18046878 PMC2695176

[ref23] Squires N. K. Squires K. C. Hillyard S. A. (1975). Two varieties of long-latency positive waves evoked by unpredictable auditory stimuli in man. Electroencephalogr. Clin. Neurophysiol. 38, 387–401. doi: 10.1016/0013-4694(75)90263-1, 46819

[ref24] Timm H. W. (1982). Analyzing deception from respiration patterns. J. Police Sci. Adm. 10, 47–51.

[ref25] Uchino B. N. Birmingham W. Berg C. A. (2010). Are older adults less or more physiologically reactive? A meta-analysis of age-related differences in cardiovascular reactivity to laboratory tasks. J. Gerontol. Psychol. Sci. 65B, 154–162. doi: 10.1093/geronb/gbp127, 20054015 PMC4499918

[ref26] Watsford M. L. Murphy A. J. Pine M. J. (2007). The effects of aging on respiratory muscle function and performance in older adults. J. Sci. Med. Sport 10, 36–44. doi: 10.1016/j.jsams.2006.05.002, 16814604

[ref27] Zaitsu W. (2016). External validity of concealed information test experiment: comparison of respiration, skin conductance, and heart rate between experimental and field card tests. Psychophysiology 53, 1100–1107. doi: 10.1111/psyp.12650, 27031043

[ref28] Zelinski E. M. Walsh D. A. Thompson L. W. (1978). Orienting task effect on EDR and free recall in three age groups. J. Gerontol. 33, 239–245. doi: 10.1093/geronj/33.2.239, 627707

